# Current Strategies in Assessment of Nanotoxicity: Alternatives to In Vivo Animal Testing

**DOI:** 10.3390/ijms22084216

**Published:** 2021-04-19

**Authors:** Hung-Jin Huang, Yu-Hsuan Lee, Yung-Ho Hsu, Chia-Te Liao, Yuh-Feng Lin, Hui-Wen Chiu

**Affiliations:** 1 Graduate Institute of Clinical Medicine, College of Medicine, Taipei Medical University, Taipei 11031, Taiwan; helix70258@gmail.com; 2 Department of Cosmeceutics, China Medical University, Taichung 406040, Taiwan; yhlee@mail.cmu.edu.tw; 3 Division of Nephrology, Department of Internal Medicine, Hsin Kuo Min Hospital, Taipei Medical University, Taoyuan City 320001, Taiwan; yhhsu@tmu.edu.tw; 4 Department of Internal Medicine, School of Medicine, College of Medicine, Taipei Medical University, Taipei 11031, Taiwan; 19386@s.tmu.edu.tw; 5 TMU Research Center of Urology and Kidney, Taipei Medical University, Taipei 11031, Taiwan; 6 Division of Nephrology, Department of Internal Medicine, Shuang Ho Hospital, Taipei Medical University, New Taipei City 23561, Taiwan; 7 Department of Medical Research, Shuang Ho Hospital, Taipei Medical University, New Taipei City 23561, Taiwan

**Keywords:** alternative animal test, nanotoxicity, cell-based test, tissue engineering, computational approach

## Abstract

Millions of experimental animals are widely used in the assessment of toxicological or biological effects of manufactured nanomaterials in medical technology. However, the animal consciousness has increased and become an issue for debate in recent years. Currently, the principle of the 3Rs (i.e., reduction, refinement, and replacement) is applied to ensure the more ethical application of humane animal research. In order to avoid unethical procedures, the strategy of alternatives to animal testing has been employed to overcome the drawbacks of animal experiments. This article provides current alternative strategies to replace or reduce the use of experimental animals in the assessment of nanotoxicity. The currently available alternative methods include in vitro and in silico approaches, which can be used as cost-effective approaches to meet the principle of the 3Rs. These methods are regarded as non-animal approaches and have been implemented in many countries for scientific purposes. The in vitro experiments related to nanotoxicity assays involve cell culture testing and tissue engineering, while the in silico methods refer to prediction using molecular docking, molecular dynamics simulations, and quantitative structure–activity relationship (QSAR) modeling. The commonly used novel cell-based methods and computational approaches have the potential to help minimize the use of experimental animals for nanomaterial toxicity assessments.

## 1. Introduction

Experimental animals are widely used as a tool to evaluate the toxicological or biological effects of potential drug candidates for the development of new treatments. To date, animal ethics and animal consciousness have been growing, resulting in these topics becoming important issues over the last decade. Unfortunately, millions of animals continue to be used in medical research for scientific purposes each year. Alternative methods of animal research have been suggested as a good strategy to avoid unethical animal procedures and make scientific experiments more humane. Therefore, the concept of the replacement of animal testing was proposed by Charles Hume and William Russell in 1957 [1]. In 1959, the 3Rs (i.e., reduction, refinement, and replacement) strategy was first discussed in the book “The Principles of Humane Experimental Technique”, which was published by two English researchers, W. M. S. Russell (1925–2006) and R. L. Burch (1926–1996) [2,3]. The principle of Russell and Burch’s 3Rs, including reduction, refinement, and replacement, aimed to reduce the number of animals used and minimize the stress to such experimental animals in medical and biological research. At present, the principle of the 3Rs is applied in laboratories where animals are used to perform more humane animal research. Implementation of the 3Rs principle has the potential to overcome the drawbacks of traditional animal tests, such as the time-consuming nature, expensive feeding requirements, and associated ethical problems [4,5]. Nowadays, alternative methods are available to replace animal tests in the majority of commonly studied biomedical fields [6,7], including cell-based cytotoxic testing, genotoxicity, and biochemical assay. Non-animal approaches, which can replace animal tests, can provide quicker, more effective, and cheaper chemical safety assessments that function as substitutes for traditional animal experiments. Alternative methods to animal tests include chemical-based tests, in vitro cell culture systems, in silico computational biomodeling, and ex vivo tests using tissue from dead animals [8]. Compared to ex vivo tests, tissue engineering is a more ethical approach to the potential replacement of animal models. Tissue is taken from dead humans or animals to perform ex vivo tests, while artificial tissue utilizes cell and material methods in tissue engineering without any possible ethical problems. In this review article, we discuss the recent applications of alternative non-animal systems, including cell-based tests, computer-based modeling, and tissue engineering, for nanotoxicity assessments in scientific experiments.

**Figure 1:**
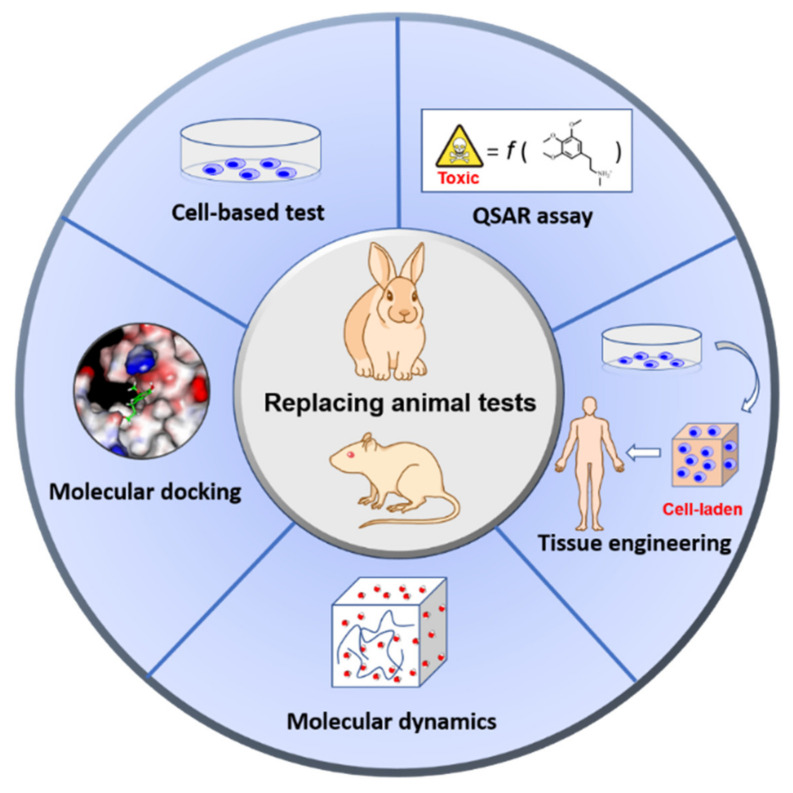
An overview of the current alternatives to the use of animals, including cell-based tests, tissue engineering, and computer-based techniques for nanotoxicity assessment. Cell-based tests and tissue engineering are in vitro approaches. In silico techniques include the application of prediction methods in nanotoxicity studies, such as molecular docking; quantitative structure–activity relationship (QSAR) assays; molecular dynamics simulations. Both in vitro and in silico approaches can be used to overcome ethical problems in medical research and nanotechnology.

The rapid growth of nanotechnology has contributed to the urgent requirement of safety assessments for manufactured nanomaterials. Therefore, analytical methods of nanotoxicity have received a great deal of attention due to the application of nanomaterials in diverse research areas, such as agriculture [9], food industries [10], medicine [11], and biotechnology [12]. Upon the development of a novel nanomaterial, an assessment of its toxic and biological impacts must be performed to understand the potential risk factors before the material can finally be applied for medical use. Nevertheless, the number of experimental animals used in nanoparticle (NP) toxicity tests has increased each year [13]. In order to reduce the use of animal testing, humane experimental techniques have been employed to investigate biological and pathological effects as alternative forms of nanomaterial toxicity assessments. In this review, we introduce that in vitro cell-based models and in silico computational models are the most commonly used alternative testing tools to evaluate the safety and toxicity of chemical substances in the field of nanotechnology (Figure 1) [14,15,16].

For in vitro experiments, cell-based tests and tissue models are widely used alternative models to determine chemical hazards. In general, in vitro studies can be applied to investigate the minimum toxic effects in a specific cellular environment via cell viability protocols [17], while in vitro methods do not represent a complete replacement for in vivo assessments. Hence, in silico methods, in the replacement of animal testing, are a useful strategy to explore the relationship between chemical and biological systems in medical product development and safety assessments. Via the pharmacokinetic information database [18], numerous in silico program tools can predict absorption, distribution, metabolism, and excretion/toxicity (ADME/T) properties, derived entirely from the structures of the chemicals of interest [19,20,21].

## 2. Category of In Vitro Models

Cell-based tests, known as in vitro techniques, are commonly used to assess the safety and toxicity of drugs and chemicals in the replacement of animal testing [22]. In vitro studies are capable of providing faster, inexpensive, and valuable information that helps researchers to assess the potential risks or possible toxicity of newly developed nanomaterials before their final application [17]. Herein, we describe the commonly used novel in vitro methods that comply with the 3Rs principle and avoid the use of animal experimentation.

### 2.1. Stem Cell Technology

Currently, cell-based assessments are utilized for the treatment of various prominent disorders—including cardiovascular, neurological, ophthalmologic, skeletal, and autoimmune disorders—and for evaluating the toxicity associated with such treatments [23]. To date, various stem cell sources exist that can be used for toxicity testing, such as fibromatosis-derived stem cells (FSCs) [24], mesenchymal stem cells (MSCs) [25], cardiac stem cells (CDCs) [26], and embryonic stem cells (ESCs) [27,28]. Human ESC research was first reported in 1998 [29], in which cells obtained from pre-implantation embryos were shown to be pluripotent in nature, with the ability to differentiate into various cell types. Thus, human ESCs may be suitable as a source of cells to be used in the development of tissue engineering [30]. Currently, several ethical and religious concerns remain within the context of using human ESCs for toxicological studies or experimentation purposes [31,32]. Human ESC research is considered an ethical problem due to the destruction of human embryos [33]. In this context, alternative stem cell sources have received a great deal of attention in order to avoid ethical quandaries for investigators [34].

Induced pluripotent stem cells (iPSCs) are multipotent stem cells that are directly obtained from skin or blood cells, which can overcome the ethical considerations related to research and publication [35]. According to the seminal report from Shinya Yamanaka’s lab in 2006 [36], the first iPSC technique used four encoding transcription factors (named Oct4, Sox2, Klf4, and C-myc) to convert reprogrammed adult mouse fibroblasts into pluripotent stem cells. The above four factors, also called the “Yamanaka factors”, have the ability to reprogram mouse or human somatic cells and differentiate them into iPSCs. The iPSCs have self-renewing properties and can differentiate into all cell types, demonstrating specific functions. Therefore, iPSCs have a great capacity for tissue repair and safety assessment applications, without the associated ethical concerns [37,38]. In the development of toxicity testing by using iPSCs, some studies have successfully incorporated iPSCs with genetic diversity into cardiotoxicity testing [39]. In a recent study, iPSCs as a cell-based in vitro model were regarded as a new approach in nanotoxicity assessment to evaluate the safety of engineered NPs [40]. For instance, hepatocyte-like cells (HLCs) induced from iPSCs have been suggested as an alternative in vitro hepatotoxicity model to study NP toxicity. The iPSC-derived HLCs can be used to assess the hepatotoxicity of silver NPs (AgNPs) [41]. Similarly, the toxicity of ZnO NPs can also be investigated by human iPSC-derived cardiomyocytes (hiPSC-CMs) for cardiac safety evaluation [42]. Thus, iPSCs not only play a role in preclinical drug toxicology studies but are also used as testing platforms for NP toxicity assessments.

### 2.2. Tissue Engineering

Tissue engineering is a multidisciplinary field that applies disciplines related to engineering, materials, medical, and biological sciences [43]. At present, tissue engineering could be used to customize the biochemical and physicochemical factors of different types of biological tissues. Scaffold-based tissue engineering is a commonly used technique in the formation of viable tissues for clinical or biological applications. For instance, chitosan-based materials represent one of the most well-known strategies for mimicking the physicochemical properties of the extracellular matrix (ECM). The advantage of using three-dimensional (3D) scaffolds is to provide a proper environment for cell adhesion and growth, which can facilitate cell differentiation associated with the replacement of biological tissues. In order to design 3D scaffolds for customization, the shape of the 3D scaffold can be established by the unique properties of smart material and 3D printing technologies. The customized shapes of 3D scaffolds provide a suitable environment for mimicking tissue [44].

The aim of this technique is to successfully produce ideal biopolymer-based 3D organ structures that can be used for NP toxicity assessments, with the eventual goal of replacing animal testing. The 3D organ structure models require the integration of synthetic or natural biological materials and stem cells for cell growth and differentiation, which provide cell-to-cell interactions and appropriate cell signaling pathways and facilitate growth in all directions during the process of cell culture. The thermally responsive properties, shape memory, and self-healing mechanisms of synthetic materials are regarded as crucial factors for approaching the design of cell-based screening systems in 3D models. Over the past decade, several studies have revealed that the synthetic materials used in tissue engineering are able to modulate stem cell differentiation via external stimulation to change their properties in 3D culture environments [45,46].

Tissue engineering has been employed to develop 3D culture environments as an alternative to animal testing toxicity assessment methods [47,48]. Traditional two-dimensional (2D) cell cultures are most commonly employed to study biological processes, mechanisms of diseases, drug development, and toxicity assays [49]. Nevertheless, 2D cultures present many limitations to mimicking in vivo conditions, such as the lack of tissue–tissue interfaces [50], presence of natural barriers, hypoxic gradients, and tight cell–cell junctions that reduce the efficiency of drug diffusion [51]. Cells grown in a 3D condition are able to mimic the natural conditions of tissues, thereby overcoming the limitations of 2D culture systems. Three-dimensional culture models can be classified into three typical types of preparation methods that are used in research studies: (i) scaffold-based techniques; (ii) cell spheroids grown on gel-like substances, and (iii) scaffold-free cell cultures in suspension [49]. Table 1 displays a comparison of the advantages and disadvantages of 2D and 3D cell culture methods. Notably, 2D cultures allow for easier cell observation and measurements, while they are also less expensive than 3D cultures.

For most fields of research, 2D cell cultures are still used in the majority of cell research studies and toxicity assessments. However, obviously, the available space in 2D cell cultures only exists in two dimensions. The limitations of 2D culture models include adhesion dependence, reduced biological environment size, and insufficient amount of cell–cell interactions. Therefore, 2D cell culture systems are not a good strategy for understanding how cells grow and function in an animal’s body. Several studies have demonstrated that cell cultures in 3D environments are more similar to in vivo cells compared to 2D cell cultures [59,60,61]. Co-culture systems can be carried out for 2D cultures as well as 3D models. These allow two or more different types of cells to be cultured on the same dish in order to study cellular interactions. However, 2D co-culture systems grow cells on a flat surface and have limited access for cell–cell contact. In contrast, 3D co-cultures can recapitulate the native cellular microenvironment. Thus, 3D co-culture system conditions are more capable of facilitating the evaluation of cell communication, cell–cell signaling, and interactions between different types of cultured cells. Three-dimensional cell culture systems can be successfully employed as an alternative to animal tests due to these systems being cost-effective and time-saving methods of tissue growth [62]. Additionally, 3D culture systems have the ability to reduce the time periods necessary for drug or toxic testing.

## 3. Category of In Silico Models

In silico modeling is a relatively new subject that combines experimental approaches, providing a powerful technique to help in understanding mechanisms at the atomic level [63,64]. Over the past decade, in the field of NP research, in silico modeling has been employed to investigate safer nanomaterials, in the replacement of animal experiments [65,66]. Since 2006, the Organization for Economic Co-operation and Development (OECD) has hosted the Working Party on Manufactured Nanomaterials (WPMN), aimed at developing appropriate strategies to ensure the safe use of nanomaterials and prevent the potential risks of nanotoxicity [67]. However, the assessment of a huge variety of different NPs is an inefficient and expensive method. Hence, in silico methods, such as bioinformatics and computational approaches, have become a comprehensive tool that allows one to evaluate the possible risks of NPs. Currently, nano-specific databases can be used to study NP risk assessments, such as the Online Chemical Modeling Environment (OCHEM), NanoDatabank, NanoHub, NanoMILE, and ModNanoTox [68]. To study NPs as new pharmaceutical drugs aimed at the prevention and treatment of diseases, the physico-chemical and structural properties of the nanomaterial must be investigated via in silico approaches. Molecular docking, quantitative structure–activity relationship (QSAR) studies, and molecular dynamics (MD) simulations are three typical categories of computational approaches that represent alternatives to animal testing in nanotoxicology studies. In this section, we summarize the various types of molecular modeling and their application in nanotoxicity assessments.

### 3.1. Molecular Docking

Molecular docking studies are a promising approach for computational simulations and the evaluation of chemical–biomolecule interactions based on 3D structural knowledge. Briefly, docking studies are simulation techniques that predict how small molecules (e.g., drugs or NPs) interact with large biomolecules (e.g., proteins or enzymes). In the first step of a docking study, all possible conformations and orientations of each ligand are generated according to the shape of the defined binding site in the protein structure. The second step involves carrying out scoring functions to approximately predict favorable interactions between the protein and the docked ligand, along with the possible orientations. After the docking study procedure, the docking scores, calculated by the scoring functions, are used to rank each properly fitted ligand in its binding site, which can then be used to define the best affinity ligand for the target protein. A good docking score means that the molecule has favorable intermolecular interactions, including hydrogen bonds, electrostatics, and hydrophobic interactions, for a given ligand and demonstrates the best affinity as a potent binder. The docking process places the rigid chemical compounds into the active site of the protein crystal structure that is obtained from the RCSB protein databank. Generally, the accuracy and speed of the docking conformation is strongly related to the types of search algorithms used. Each docking application is based on a conformational search algorithm, such as the genetic algorithm (GA) [69], Monte Carlo (MC) [70], and incremental construction (IC) [71].

A target protein interacts with the docked ligand and further generates a protein–ligand complex which may enhance or inhibit the biological function in the experimental test. In the past few years, the molecular docking technique has been applied to predict the binding affinity of bioactive compounds at the specific site of a target protein for studying the protein–ligand interactions. Recently, some studies have employed docking methods to analyze the binding conformations of ligands for toxicity assessments as alternatives to using animal models. Hence, the docking strategy can be applied to study the chemical interactions of NPs with target enzymes. This has provided insights into the possible mechanism from which the geometric structures of the protein–NP complex are formed.

Thus far, computational modeling has utilized the protein crystal structure to investigate the cytotoxic effects and potential risks associated with functionalized NPs in many fields of nanotechnology [72,73,74,75]. For instance, cytochrome P450 (CYP) is an important enzyme system for drug metabolism. Hence, molecular docking approaches can be applied to NP and CYP enzymes in order to estimate the molecular interactions of the protein–ligand complex on the basis of its 3D structure [76]. Inhibition of the CYP enzyme by co-administered drugs is clinically significant in terms of drug−drug interactions, which may reduce drug efficacy or increase toxicity [77,78,79]. In silico simulations provide insights into the potential mechanism of the inhibitory effects on the CYP enzyme after interaction with NPs. Additionally, these models can be used in collaboration with in vitro experiments for estimating toxicological properties in the development of novel nanomaterials. As another example, molecular docking has also been used to determine the potential toxicity of different types of NPs with biological macromolecules, including CuO, TiO_2_, Fe_3_O_4_, Au, Ag, ZnO, Mn_2_O_3_, and Fe_3_O_4_ [80]. Hence, docking analysis between NPs and biological molecules has gained increased attention in the field of nanotoxicology as an alternative method that is able to predict potential toxicity [81].

### 3.2. QSAR Assay

The QSAR assay—a computer-based technique—is a promising tool that can be used as an alternative method to replace animal tests in toxicity prediction. Regarding the QSAR model, biological activity or toxic effects are displayed as a function that is related to various types of molecular descriptors, as follows:

Biological activity/toxicity = ***f*** (molecular descriptors) (1)

**Figure 2:**
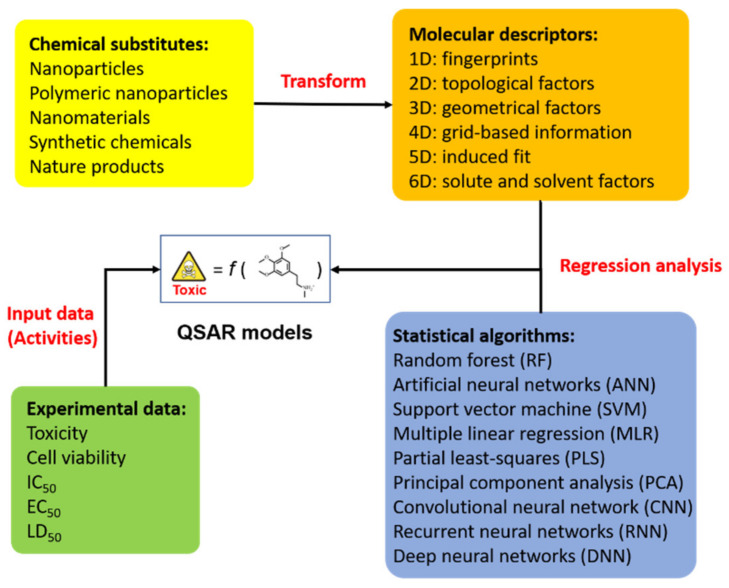
A schematic overview of QSAR model generation in the assessment of chemical substitutes and prediction of their toxicity. The schematic diagram illustrates a typical method based on different statistical algorithms and specific molecular descriptors for building a predictive regression model. QSAR modeling could be employed to combine experimental measurements for in silico prediction in drug design or nanotoxicology research.

The computational technique helps to reduce the costs associated with labor, resources, and time in nanotoxicity assessments [82]. QSAR modeling is an efficient method of predicting the biological activity or toxicity of chemical substances on the basis of mathematical statistics and knowledge of machine learning (Figure 2). The concept of QSAR was proposed a century ago, and Crum-Brown and Fraser demonstrated that the composition of chemicals is associated with their physiological activity [83]. Following this, simple chemical compounds in response to the free energy relationship model were first proposed by Corwin Hansch in 1962 [84]. The basic aim of the QSAR model is to define an appropriate function that has a reasonable relationship between the chemical structure and biological activity. This has the potential to further summarize the physio-chemical and biological information in order to predict toxicity effects or develop ideal nanomaterials.

According to the dimensions of molecular descriptors used for model generation, QSAR methods can be classified into several classes of modeling, such as 1D, 2D, 3D, 4D, 5D, and so forth [85]. In most cases, 2D- and 3D-QSAR studies are commonly used to evaluate the series of chemical compounds [86,87,88]. The 1D-QSAR approach allows for the determination of correlations for 1D descriptors (pKa, log P, structural fragments, and fingerprints) with biological activity [89]. To date, the 2D-QSAR method has been widely explored in many studies for the study of toxic or medicinal chemistry, which was first proposed in the early 1970s [90]. In general, the physio-chemical properties of 2D-QSAR consider various parameters, including geometric parameters, the polar surface area, topological indices, and molecular fingerprints. In the process of model generation, 2D-QSAR correlates the above physio-chemical properties with biological activity, which can be applied to identify the predictive toxicology for organic compounds [91]. However, the lack of steric properties is a disadvantage of 2D-QSAR. To solve this problem, the 3D-QSAR method employs the 3D properties (steric and electrostatic fields) surrounding the interesting molecule and uses chemometric methods to build a relationship between the structural properties and the molecule’s activity [92].

As for the statistical methods used for QSAR models, the correlation technique is used to construct relationship models between structural information and biological activity/toxicity. According to the different types of machine learning algorithms used, QSAR generation can be classified into two major categories: linear and non-linear methods. The linear regression methods contain principal component analysis (PCA), multiple linear regression (MLR), and partial least-squares (PLS) methods. The non-linear regression algorithms consist of random forest (RF), support vector machine (SVM), and artificial neural networks (ANNs). For instance, 3D-QSAR employs statistical methods such as the ANN algorithm, PLS analysis, PCA methods, and cluster analysis for model generation. Therefore, the predictive power of 3D-QSAR makes it more suitable than 2D-QSAR [93]. Recently, artificial intelligence (AI) has been used as a statistic algorithm to search for potential regression models during the process of QSAR model generation. For example, the deep-learning-based QSAR model employs a convolutional neural network (CNN), recurrent neural networks (RNNs), and deep neural networks (DNNs) to optimize the quality of predictive models.

QSAR methods have been applied in many scientific disciplines. For instance, the QSAR approach is frequently used in risk assessments, toxicology, regulatory decision making, and chemical regulation. Thus, in silico techniques have the potential to replace animal tests on the basis of their ability to statistically and reliably predict the likelihood of a chemical substance being hazardous. Such in silico techniques replicate similar conditions to those existing in human biology. Table 2 shows various different QSAR modeling studies that are used to understand the nanotoxicity of chemical substitutes, such as NPs, metal oxide, and fullerenes. In some countries, governments utilize QSAR tools for toxic hazard prediction to avoid animal testing [94]. In recent years, researchers have applied QSAR models to analyze potential toxic effects involved in the manufacturing of nanomaterials [93].

### 3.3. MD Simulation

MD simulation is a mature method and is widely used to investigate chemical and physical properties in the modern era of computational nanotoxicology. MD simulations can be regarded as a complementary technique to examine the atomic motion of 3D structures that are obtained from experimental data, such as X-ray crystallography and nuclear magnetic resonance (NMR) spectrometry. Modern MD simulations not only assist with understanding the time-dependent behavior of the physical movements of atoms and molecules but also provide the thermodynamic and kinetic properties of the material systems at the atomic level. Therefore, detailed information on the conformational changes of macromolecules and NPs can be observed from MD simulations.

Computational methods, by using MD simulations, can be employed to generate toxicity prediction models that can be used in NP design and development. Toxicity assessments using traditional cell-based or animal tests have ethical considerations, financial problems, and are time-consuming. Hence, computational toxicology is widely applied in biomedical research to estimate toxic effects on various biological systems. Hence, this method is an alternative strategy for studying the toxicity of chemical compounds. In a recent study, MD simulations were applied to test various hypotheses of toxicity mechanisms in the field of computational toxicology and nanotoxicology. For instance, the aggregation and agglomeration of NPs are responsible for increased toxicity during the process of preparation or in the polymer matrix [102]. Consequently, this can induce apoptosis and generate intracellular reactive oxygen species (ROS) [103]. For example, agglomeration of titanium dioxide (TiO_2_) NPs was shown to trigger toxicity responses in cell-based and animal experiments. Meanwhile, large agglomerates of TiO_2_ NPs facilitated stronger biological effects, such as DNA damage, GSH depletion, and inflammation, compared to small agglomerates [104]. Therefore, studying the aggregation of multiple NPs is an important strategy to investigate their toxic effects.

## 4. Challenges of Alternative Testing Strategy

The major drawback of in vitro methods is the failure to mimic in vivo situations. For instance, in vitro models have no intrinsic circulation associated with the drug delivery system [105]. The toxicity effects of chemicals were not represented in the affected tissues. Additionally, chronic effects cannot be tested via in vitro studies due to difficulties in the consequences of long-term simulation [106]. Actually, in vitro test systems have limitations in establishing in vivo-relevant systems. In some cases, as the isolated primary cells have difficulty corresponding to the relevant cell type in biological organs, it may not easy to construct reasonable in vitro models corresponding to in vivo testing models. In addition, there are still problems in estimating the in vitro concentrations with regard to in vivo doses. In most research, in vitro alternative testing is hard to extrapolate from cellular pathways to in vivo toxicological outcomes [107].

For in silico computational approaches, assessing the accuracy of in silico prediction is still a major challenge for nanoparticles. For instance, NPs have different particle sizes, surface properties, aggregate sizes, and solubility influencing their toxicity [108]. Hence, there are still restrictions to identify the accurate physicochemical properties of NPs to predict reasonable hazards. However, the use of NPs in cytotoxicity assays presents drawbacks in common toxicity methods, such as water-soluble tetrazolium salts (WSTs), propidium iodide, and oxidative stress detection assays, due to optical interference. During the process of optical detection, the NPs may have the same spectral range as absorbing light and, thus, lead to incorrect results [108].

Therefore, the development of in vitro and in silico tests involves technical challenges to set up reliable culture conditions with in vivo-like levels to perform hazard testing of NPs. The use of non-mammalian alternative models may be an ideal strategy to overcome the ethical concerns related to the traditional animal models in the safety assessment of NPs. To assess the hazards of engineered NPs, non-mammalian models such as [109] *Caenorhabditis elegans* (*C. elegans*) [110], Drosophila (*Drosophila melanogaster*) [111], African clawed frog (*Xenopus laevis*) [112], chicken chorioallantoic membrane (*Gallus gallus*) [113], and zebrafish (*Danio rerio*) [114] could be considered as reliable approaches to improve the reliability of assessments of the toxicity of NPs.

## 5. Conclusions

Toxicity assessment allows us to explore the toxic effects of developed chemical substitutes, such as NPs and nanomaterials. However, animal consciousness and ethics are important issues to be considered during the process of manufacturing nanomaterials. Implementation of the 3Rs principle has facilitated the development of various alternative methods that have been suggested in the replacement of traditional animal tests. The use of analytical techniques, including in vitro cell-based assays, tissue engineering, in silico structure-based techniques, and QSAR studies, can lead to the avoidance of unethical experiments. The alternative methods can not only be used to study nanotoxicity but can also be extensively used in drug design and applied in clinics. In addition, in silico modeling could also combine in vitro experimental approaches for providing a powerful technique to investigate the mechanism for newly developed products. It is important to establish the relationship between the physicochemical characteristics of NPs and nanotoxicity. Nevertheless, achieving accurate toxicological results of NPs is dependent on factors such as particle size, surface properties, aggregate size, and solubility. Therefore, the prediction of possible nanotoxicity requires systemic examination to understand the molecular and cellular mechanisms involved. Nanotoxicity assays should utilize multidisciplinary approaches, including in vitro and in silico experimental models, to avoid contradictory results. These current strategies for alternatives to animal testing are regarded as important approaches that are necessary to meet the 3Rs principle, which will result in reducing the number of experimental animals used in the future.

## Figures and Tables

**Table 1:** The advantages and disadvantages of 2D and 3D cell culture models.

Characteristics	2D Culture	3D Culture	Reference
Cell growth rate	The growth rate faster than in vivo test	The growth rate depends on the cell type	[52]
Quality of cell growth	Long-term and easy to culture	Long-term and easy to culture	[53]
Sub-culturing time	About 1 week	Up to 4 weeks	[54]
Cell–cell interactions	Lack of space for cell–cell or cell–ECM interactions	More available space to provide proper cell–cell or cell–ECM interactions	[55]
Cost of preparation for cell culture	Low-cost maintenance	More expensive and time-consuming	[56]
In vivo mimics	Limitation of imitating the natural organs	Natural structures are 3D	[57]
Preparation of cell culture	A few hours	From hours to many days	[58]

**Table 2:** Main approaches of QSAR models for nanotoxicity evaluation.

Statistical Algorithm	Chemical Substitute	Statistical Software	Measurement	Reference
Random forest	Metal oxide	R	Cell viability	[95]
Artificial neural network	Carbon nanotubes, fullerenes, and silica NPs	CORAL	Cytotoxicity	[96]
Support vector machine	Q-dots and FeOx NPs	R and Python	Cellular uptake	[97]
Genetic algorithm and multiple linear regression	Thiol-gold NPs	TREOR	Cell viability	[98]
Partial least-squares	SiO_2_, TiO_2_, CeO_2_, AlOOH, ZnO, Ni(OH)_2_	R	Cytotoxicity	[99]
Deep neural network and k-nearest neighbor	Q-dots and FeOx NPs	R	Cellular uptake	[100]
Bayesian networks	NPs	Python	Cytotoxicity	[101]
